# Human post‐infection serological response to the spike and nucleocapsid proteins of SARS‐CoV‐2

**DOI:** 10.1111/irv.12798

**Published:** 2020-08-25

**Authors:** Cheng Xiao, Shiman Ling, Minshan Qiu, Zhenxuan Deng, Liping Chen, Airu Zhu, Yi Chen, Yong Liu, Xia Lin, Fangmei Lin, Qiubao Wu, Lihan Shen, Feng Ye, Xiaoqing Liu, Yimin Li, Jincun Zhao, Zifeng Yang, Benjamin J. Cowling, Richard Webby, Mark Zanin, Sook‐San Wong

**Affiliations:** ^1^ Guangzhou Medical University Guangzhou China; ^2^ State Key Laboratory of Respiratory Disease National Clinical Research Center for Respiratory Disease Guangzhou China; ^3^ Department of Intensive Care Dongguan People's Hospital Dongguan China; ^4^ Central Laboratory Guangzhou Women and Children's Medical Center Guangzhou Medical University Guangzhou China; ^5^ Kingmed Virology Diagnostics and Translational Center Guangzhou Kingmed Center for Clinical Laboratory Guangzhou China; ^6^ Guangzhou Institute of Respiratory Health The First Affiliated Hospital of Guangzhou Medical University Guangzhou China; ^7^ Nanshan Medicine Innovation Guangdong China; ^8^ Department of Pulmonary and Critical Care Medicine The First Affiliated Hospital of Guangzhou Medical University Guangzhou China; ^9^ Department of Intensive Care The First Affiliated Hospital of Guangzhou Medical University Guangzhou China; ^10^ Macau University of Science and Technology Macau SAR China; ^11^ School of Public Health The University of Hong Kong Hong Kong SAR China; ^12^ Division of Virology St. Jude’s Children’s Research Hospital Memphis TN USA

**Keywords:** antibodies, epidemiology, nucleocapsid, SARS‐CoV‐2, serology, spike

## Abstract

To inform seroepidemiological studies, we characterized the IgG‐ responses in COVID‐19 patients against the two major SARS‐CoV‐2 viral proteins, spike (S) and nucleocapsid (N). We tested 70 COVID‐19 sera collected up to 85 days post‐symptom onset and 230 non‐COVID‐19 sera, including 27 SARS sera from 2003. Although the average SARS‐CoV‐2 S and N‐IgG titers were comparable, N‐responses were more variable among individuals. S‐ and N‐assay specificity tested with non‐COVID‐19 sera were comparable at 97.5% and 97.0%, respectively. Therefore, S will make a better target due to its lower cross‐reactive potential and its' more consistent frequency of detection compared to N.

## 1 INTRODUCTION

Seroepidemiological studies are urgently needed to understand the true incidence of coronavirus disease 2019 (COVID‐19) and gauge the level of population immunity. Since such studies typically involve screening a large number of sera, the assays of choice should be highly sensitive, high‐throughput, and safe. Enzyme‐linked immunoassays (EIA), particularly those using recombinant proteins, although less specific than neutralization assays, have the advantage of being high‐throughput, safe, and easy to standardize.

The major antibody responses after coronavirus infections are directed against the Spike (S) and Nucleocapsid (N) proteins.^1^ During Severe Acute Respiratory Syndrome Coronavirus‐1 (SARS) infections, anti‐N antibodies appeared earlier and were subject to higher cross‐reactivity than anti‐S antibodies, while anti‐S antibodies were better correlated to neutralization activity.^2^ Aside from the zoonotic origin SARS and Middle‐East Respiratory Syndrome (MERS) coronavirus, there are four human strains of coronaviruses (reviewed in^3^) and seroprevalence to any of these viruses in older adults can be greater than 90%.^4^ Thus, the COVID‐19 serological assays should be sensitive and specific enough to discriminate responses from other coronaviruses.

As there are a number of commercial kits available to detect SARS‐CoV‐2 antibodies based on the S and N proteins, we wanted to understand the longitudinal kinetics of the COVID‐19 antibody responses to both proteins and the specificity of the S‐ and N‐ELISA‐based assays to facilitate future seroepidemiological studies.

## 2 MATERIALS AND METHODS

### 2.1 Ethics statement

The ethics committee of The FAHGMU (Ethics No. 2020‐85) and Dongguan's People's Hospital (KYKT2020‐005‐A1) has approved the use of patient's samples for this study.

### 2.2 Serum source

Seventy sera collected between days 0 and 85 post‐symptom onset were obtained from 31 laboratory‐confirmed COVID‐19 cases (aged 26 to 82 years old, median = 58) admitted to The First Affiliated Hospital of Guangzhou Medical University (FAHGMU) (N = 18) and Dongguan People's Hospital (N = 13). Patients were confirmed infected based on positive nucleic acid testing according to China's National Guidelines.

The non‐COVID‐19 sera panel consist of sera from 80 healthy elderly (between 60 to 89 years old) that were collected in 2015,^5^ 28 adults and 30 children with laboratory‐confirmed influenza at FAHGMU and 35 adults and 30 children that submitted sera for non‐respiratory illness testing at an independent clinical diagnostic laboratory. Thirty archived sera from patients infected with SARS‐CoV during the 2003 outbreak in Guangdong were screened for activity, and 27 were included in our study.

### 2.3 SARS‐CoV Spike and Nucleocapsid IgG ELISA

The archived SARS sera were tested for SARS‐CoV spike (S_c_) and nucleocapsid (N_c_)‐specific IgG antibodies using an ELISA kit that was provided by Autobio Diagnostics Co. Ltd (Zhengzhou, China).

### 2.4 SARS‐CoV‐2 Spike and Nucleocapsid IgG ELISA

Recombinant SARS‐CoV‐2 S (encompassing the extracellular domain, S1 and S2 subunits) and N proteins (Sino Biological Inc, China) were used to coat 96‐well plates at 0.5μg/ml overnight at 4°C. After washing and blocking, serially diluted sera (at a starting dilution of 1:100) were added to the plate and incubated for 2 hours at 37°C. Plates were washed and added with an anti‐human IgG horseradish peroxidase‐conjugated secondary antibody (Sigma). Colorimetric reaction was developed using 3,3′,5,5′‐Tetramethylbenzidine (TMB) substrate (Gcbio Technologies, China), stopped using 0.5 mol/L sulfuric acid and the absorbance read at 450 nm. Endpoint titers were determined to be the last reciprocal dilution with a positive/negative optical density (O.D) ratio ≥2.

### 2.5 Data analyses

Continuously variable data were log‐transformed, and geometric mean titers were used to describe the average titers. Differences between groups or time points were analyzed by ANOVA. Correlations between antibody titers were tested using Pearson's correlation test, with *P*‐values of <.05 considered statistically significant. All graphs and statistical testing were performed using Prism version 8 (GraphPad Software).

## 3 RESULTS

### 3.1 Kinetics and cross‐reactivity of the antibody responses to SARS‐CoV‐2 S and N proteins in laboratory‐confirmed COVID‐19 patients

**Figure 1:**
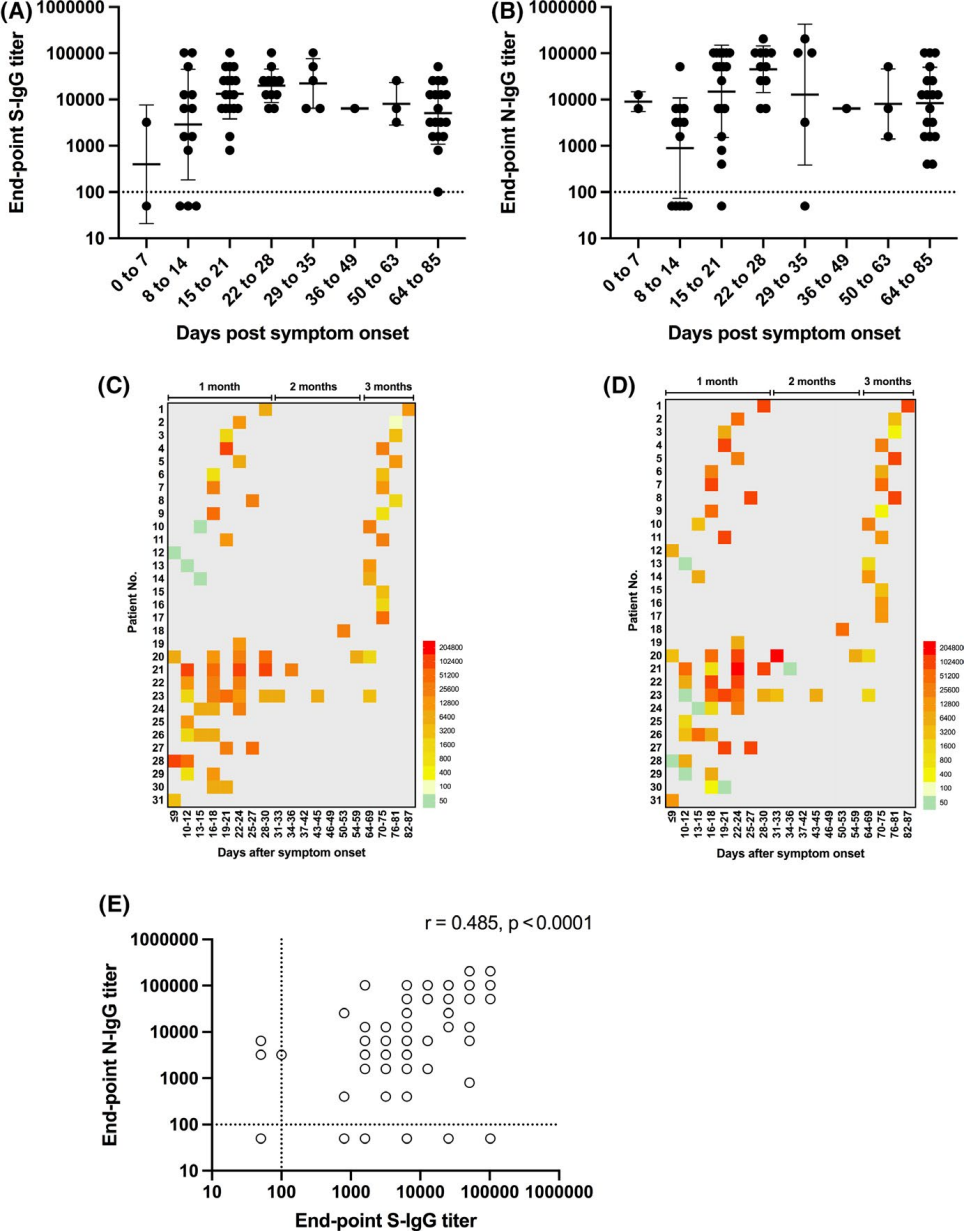
Kinetics of antibody responses against SARS‐CoV‐2 spike, S and nucleocapsid, N proteins in laboratory‐confirmed COVID‐19 patients. Endpoint IgG titers against the SARS‐CoV‐2 (A) spike, S and (B) nucleocapsid, N protein at different times post‐symptom onset in COVID‐19 sera. Individual endpoint IgG titers against the (C) S and (D) N protein at different times post‐symptom onset. (E) Correlation between S‐ and N‐IgG titers were tested by Pearson's correlation, with *P* < .05 considered significant

Within the first 2 weeks of symptom onset, N‐ and S‐IgG were both present above detection threshold in 7 of the 15 (47%) sampled sera (Figure 1A,B, Table S1). By the third week, however, S‐specific IgG titers were detected in 100% of sera, compared to 94% in which N‐specific IgG titers were detected. Of the 55 sera samples that were collected after 2 weeks of symptom onset, 100% had detectable S‐specific IgG titers and 96% had detectable N‐specific IgG titers. The average S‐ and N‐IgG titers peaked around week four (day 22 to 28), with the average titer against N being slightly higher than S and decreased by 0.6 and 0.7 logs, respectively, by week 12. There was greater variability in the N‐specific IgG response, as evident in the higher coefficient of variation (CV) for the binned N‐specific IgG titers compared to the S‐specific IgG titers (Table S1). Eleven of the ICU patients from the FAHGMU have previously been reported^6^ to have higher ratios of N/S‐specific IgG compared to mild patients within the first 2 weeks post‐symptom onset. This was also observed by week four in our study (Figure 1C,D), although this difference was no longer apparent by week 12, suggesting that the antibody dynamics may have changed according to clinical severity. Overall, there was a moderate correlation (*r* = .485, *P* < .0001) between S‐ and N‐ specific IgG titers (Figure 1E).

### 3.2 Cross‐reactivity between SARS‐CoV‐1 with SARS‐CoV‐2 antigens

**Figure 2:**
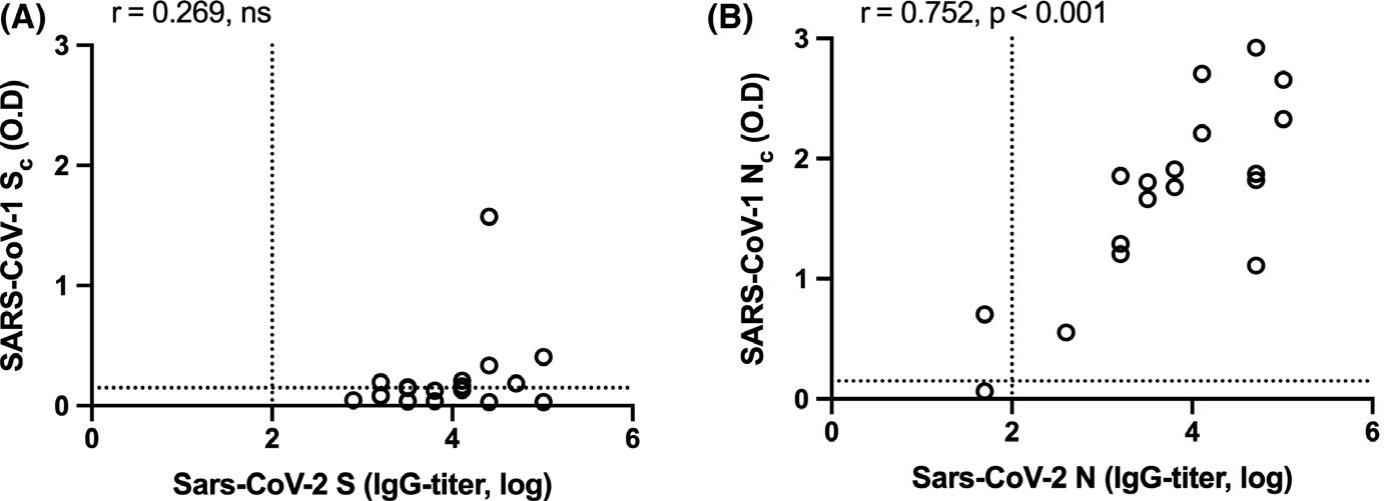
Correlation between COVID‐19 antibody reactivity to SARS‐CoV and SARS‐CoV‐2 proteins. The reactivity of 18 sera obtained from COVID‐19 patients against (A) the SARS‐CoV‐2 spike (S) and the SARS‐CoV spike (S_c_) proteins, and (B) the SARS‐CoV‐2 nucleocapsid (N) and the SARS‐CoV nucleocapsid (N_c_) proteins are shown. Log‐transformed IgG titers are shown on the Y‐axes and the optical density (O.D) values measured in the ELISAs are shown on the X‐axes. Correlation between spike and nucleocapsid IgG titers was tested by Pearson's correlation, with *P* < .05 considered significant

Since the S and N proteins of SARS‐CoV‐2 and SARS‐CoV‐1 share 77% and 94% sequence homology, respectively,^7^ we examined the cross‐reactivity between COVID‐19 and SARS sera against both these proteins. We selected 19 COVID‐19 sera to test its reactivity against the SARS‐S_c_ and SARS‐N_c_ protein. We plotted the endpoint SARS‐CoV‐2 S or N‐IgG titers against O.D value when measured using the SARS‐S_c_ and SARS‐N_c_ ELISA kits. There was a poor correlation (*r* = .269) between the S‐IgG titer and the S_c_‐O.D readout (Figure 2A). In contrast, there was a good correlation between the N‐IgG titer and the N_c_–O.D (*r* = .703, *P* < .001) readout (Figure 2B), suggesting that N is antigenically more conserved between the two viruses compared to S.

**Figure 3:**
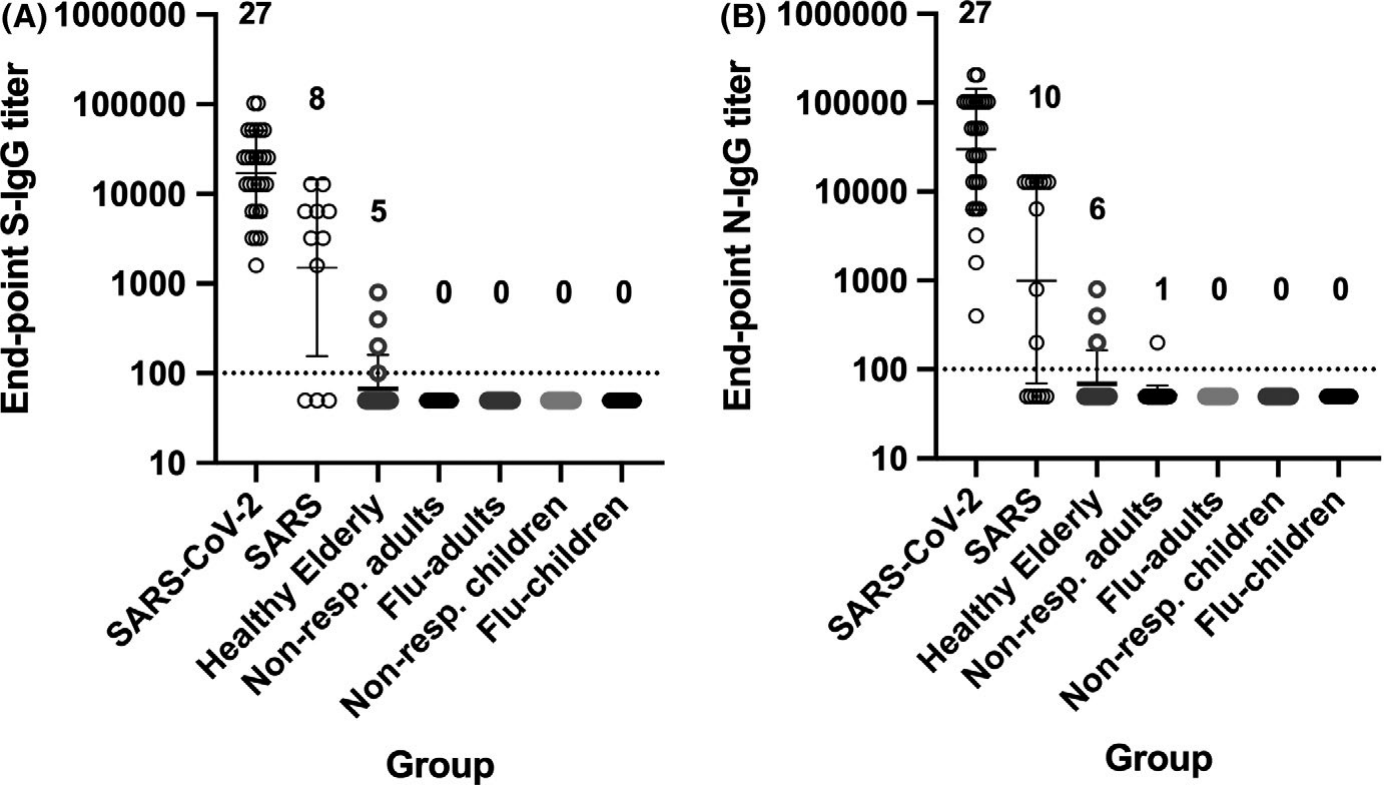
Cross‐reactivity of non‐COVID‐19 sera. Endpoint IgG titers against the SARS‐CoV‐2 spike (A) and nucleocapsid (B) proteins detected in non‐COVID‐19 serum samples. The single highest titer in COVID‐19 sera collected after day 14 post‐symptom onset (N = 27) were included for reference. For the SARS group, only SARS sera that were positive for its homologous protein were tested, that is 11 SARS‐S_c_‐positive sera were tested against SARS‐CoV‐2 S, and 16 SARS‐N_c_ positive were tested against SARS‐CoV‐2 N. Other groups consisted of: 80 healthy elderly, 35 adults (non‐respiratory testing), 28 influenza‐confirmed adults, 30 children (non‐respiratory testing), and 30 influenza‐confirmed children. Number of positive samples per total sera are indicated above the graphs

In a reciprocal manner, of the 30 SARS sera we retrieved, we confirmed the presence of SARS‐CoV spike (S_c_) and nucleocapsid (N_c_)‐specific IgG antibodies in 11 and 16 samples, respectively. The remaining three samples were negative for both SARS‐S_c_ and SARS‐N_c_. In the 11 SARS sera that were IgG‐S_c_‐positive, eight (73%) sera had IgG titers to SARS‐CoV‐2 S that were between 1,600 and 12,800. In the 16 SARS sera that were IgG‐N_c_‐positive, 10 (63%) sera had IgG titers between 800 and 12,800 to SARS‐CoV‐2 N protein (Figure 3, Table S1). This suggest that SARS sera will cross‐react with SARS‐CoV‐2 S and N proteins.

### 3.3 Cross‐reactivity of non‐COVID‐19 antibody responses

We next examined the potential for non‐specific cross‐reactivity in non‐COVID‐19 sera to SARS‐CoV‐2 S and N with. Five elderly (2.5%) samples had detectable titers against S while six samples (2.6%, five elderly and one adult) and had titers against N (Figure 3, Table S1). Excluding SARS sera, the specificity of the S‐ and N‐based ELISAs was 97.0% and 97.5%, respectively.

## 4 DISCUSSION

Studies on the antibody kinetics to SARS‐CoV‐2 have generally focused on responses within 3 weeks of symptom onset, with the longest time examined being 50 days post‐symptom onset. In our study, we found that S‐ and N‐IgG peaked at week four and were detectable at comparable titers in most COVID‐19 patients up to 3 months post‐symptom onset. This observation is similar to that observed in SARS and MERS patients,^2^, ^9^ although in some studies N‐specific IgG appeared earlier compared to S.^10^, ^11^ Follow‐up studies with MERS and SARS‐CoV patients showed that antigen‐specific IgG remain detectable in most patients for up to 3 years, with the likelihood of positivity correlating well with the initial disease severity.^12^, ^13^ Similarly, a challenge study with the human coronavirus 229E also showed that virus‐specific IgG and IgA peak by week three post‐inoculation.^14^ Taken together, coronavirus‐specific IgG can be reliably detected by week three after symptom onset.

A limitation to our study was that our COVID‐19 sera were from severely ill patients who were hospitalized and not discharged due to continued positivity for viral RNA as per national guidelines. The kinetics of responses reported here may not reflect the responses of patients with less severe infections, since the magnitude and profile of antibody responses can be influenced by disease or infection severity.^6^, ^15^, ^16^ Indeed, some asymptomatic COVID‐19 cases do not have detectable antibody responses even a month after confirmation of infection.^8^, ^17^ Thus, the association between the severity of infection and the stability of the ensuing antibody response should be systematically studied to enable accurate interpretation of seroprevalence data. Another limitation is that we were not able to analyze the degree of cross‐reactivity and non‐specificity due to human coronaviruses (hCoV). To compensate for this, we screened a larger number of non‐COVID‐19 sera with an unclear history of hCoV infection and detected SARS‐CoV‐2 S‐ and N‐IgG in a small proportion of individuals. Our assay specificity appears to be in the range of other published studies, suggesting minimal cross‐reactivity with human coronaviruses.^18^, ^19^, ^20^ Although this specificity range might still present a problem in areas of low disease prevalence, a hierarchical testing approach that includes a secondary validation test, such as neutralization assays, can be adopted to achieve both sensitivity and specificity.^15^, ^21^ Despite the comparable titers and assay specificity, our data indicate that S will make a better target due to its lower cross‐reactive potential and its slightly more consistent frequency of detection compared to N. Furthermore, its functional importance ^15^, ^19^, ^22^ will add value in using it as a serological target in any population studies.

## CONFLICT OF INTEREST

All authors declare no conflict of interest.

## AUTHOR CONTRIBUTIONS

**Cheng Xiao:** Data curation (lead); Formal analysis (lead); Investigation (lead); Methodology (lead); Supervision (lead); Writing‐original draft (supporting); Writing‐review & editing (supporting). **Shiman Ling:** Data curation (lead); Formal analysis (lead); Investigation (lead); Methodology (lead); Resources (lead); Writing‐original draft (supporting); Writing‐review & editing (supporting). **Minshan Qiu:** Investigation (supporting); Resources (supporting); Writing‐review & editing (supporting). **Zhenxuan Deng:** Investigation (supporting); Resources (supporting); Writing‐review & editing (supporting). **Liping Chen:** Investigation (supporting); Resources (supporting); Writing‐review & editing (supporting). **Airu Zhu:** Investigation (supporting); Resources (supporting); Writing‐review & editing (supporting). **Yi Chen:** Investigation (supporting); Methodology (supporting); Resources (supporting); Writing‐review & editing (supporting). **Yong Liu:** Investigation (supporting); Resources (supporting); Writing‐review & editing (supporting). **Xia Lin:** Investigation (supporting); Methodology (supporting); Resources (supporting); Writing‐review & editing (supporting). **Fangmei Lin:** Investigation (supporting); Methodology (supporting); Writing‐review & editing (supporting). **Qiubao Wu:** Investigation (supporting); Resources (supporting); Writing‐review & editing (supporting). **Lihan Shen:** Investigation (supporting); Resources (lead); Writing‐review & editing (supporting). **Feng Ye:** Investigation (supporting); Resources (lead); Supervision (supporting); Writing‐review & editing (supporting). **Xiaoqing Liu:** Investigation (supporting); Resources (lead); Supervision (supporting); Writing‐review & editing (supporting). **Yimin Li:** Investigation (supporting); Resources (lead); Supervision (supporting); Writing‐review & editing (supporting). **Jincun Zhao:** Investigation (supporting); Resources (lead); Supervision (supporting); Writing‐review & editing (supporting). **Zifeng Yang:** Investigation (supporting); Resources (lead); Supervision (supporting); Writing‐review & editing (supporting). **Benjamin Cowling:** Investigation (supporting); Methodology (supporting); Resources (lead); Writing‐review & editing (supporting). **Richard John Webby:** Conceptualization (supporting); Methodology (supporting); Writing‐original draft (supporting); Writing‐review & editing (supporting). **Mark Zanin:** Conceptualization (lead); Formal analysis (lead); Investigation (lead); Methodology (lead); Project administration (lead); Resources (equal); Supervision (equal); Writing‐original draft (lead); Writing‐review & editing (lead). **Sook‐San Wong:** Conceptualization (lead); Formal analysis (lead); Funding acquisition (lead); Investigation (lead); Methodology (lead); Project administration (equal); Resources (equal); Supervision (equal); Writing‐original draft (lead); Writing‐review & editing (lead).

## AUTHOR CONTRIBUTIONS

SSW, MPZ, and RW: Conceiving and designing of the study. CX, SML, XL, and FML: Laboratory assays and analysis of the data. AZ, LC, MQ, ZD, QW, YC, RL, LS, FY, BC, YL, JZ, and YZ: Collecting and processing of the clinical samples. SSW, MPZ, and RW: Writing of the manuscript.

## Supporting information

Table S1
