# Supplementary material for: Human post‐infection serological response to the spike and nucleocapsid proteins of SARS‐CoV‐2
Source: Influenza Other Respir Viruses. 2020 Aug 25;15(1):7–12. doi: 10.1111/irv.12798 (PMC7461388; doi:10.1111/irv.12798)
Supplement: Supplementary file 1 — Table S1 [file IRV-15-7-s001.docx]

**Supplemental Table**

**Supplemental Table 1. Details and descriptive statistics of SARS-CoV-2 spike and nucleocapsid IgG-titers in the COVID-19 and non-COVID-19 sera.** Endpoint-IgG titers were log-transformed and the average expressed as geometric means. Data dispersion was expressed as geometric standard deviations (SD) and coefficients of variation.

| **Group (N)** | **Samples** | **Time of collection** | **Number of sera** | **S** | | | | **N** | | | |
| --- | --- | --- | --- | --- | --- | --- | --- | --- | --- | --- | --- |
|  |  |  |  | **No. seropositive^†^ (%)** | **Geo Mean** | **Geo SD Factor** | **CV** | **No. seropositive^†^ (%)** | **Geo Mean** | **Geo SD Factor** | **CV** |
| **COVID-19 (N=31)** | ≤ 7 days | 7 February to 7 April 2020 | 2 | 1 (50) | 2.44 | 1.67 | 49.1 | 2 (100) | 3.95 | 1.06 | 5.4 |
|  | 8 to 14 days |  | 13 | 10 (77) | 3.23 | 1.50 | 34.6 | 8 (62) | 2.75 | 1.45 | 36.8 |
|  | 15 to 21 days |  | 18 | 18 (100) | 4.09 | 1.15 | 13.1 | 17 (94) | 4.03 | 1.35 | 24.0 |
|  | 22 to 28 days |  | 11 | 11 (100) | 4.29^*^ | 1.09 | 8.4 | 11 (100) | 4.63^*^ | 1.12 | 10.8 |
|  | 29 to 35 days |  | 5 | 5 (100) | 4.32 | 1.13 | 12.4 | 4 (80) | 3.80 | 1.62 | 37.0 |
|  | 36 to 49 days |  | 1 | 1 (100) | 3.81 | 1 | 0 | 1 (100) | 3.81 | 1 | 0 |
|  | 50 to 63 days |  | 3 | 3 (100) | 3.90 | 1.12 | 11.8 | 3 (100) | 3.86 | 1.21 | 19.3 |
|  | 64 to 85 days |  | 17 | 17 (100) | 3.65^‡^ | 1.23 | 18.5 | 17 (100) | 3.89^§^ | 1.23 | 19.6 |
| **Non-COVID-19 sera** | SARS Adults | 2003 | 27 | 8 (30) | 3 | 1.45 | 30.9 | 10 (27) | 2.77 | 1.52 | 38.4 |
|  | Healthy Elderly | 2015 | 80 | 5 (6.3) | 1.73 | 1.09 | 10.5 | 5 (6.3) | 1.73 | 1.09 | 11.4 |
|  | Non-respiratory testing adults | Mar-20 | 35 | 0 (0) | 1.69 | 1 | 0 | 1 (2.9) | 1.71 | 1.05 | 5.9 |
|  | Influenza-confirmed adults | June to July 2017 | 28 | 0 (0) | 1.69 | 1 | 0 | 0 (0) | 1.69 | 1 | 0 |
|  | Non-respiratory testing children | December 2019 | 30 | 0 (0) | 1.69 | 1 | 0 | 0 (0) | 1.69 | 1 | 0 |
|  | Influenza-confirmed children | December 2019 | 30 | 0 (0) | 1.69 | 1 | 0 | 0 (0) | 1.69 | 1 | 0 |

**^†^** Seropositive is defined as IgG-titers above the threshold of detection of 1:100 serum dilution. Titer below the detection threshold is arbitrarily set at 50.

* p< 0.01 for S vs. N-titers, unpaired t-test

^‡^ p<0.05, for titers at 64 to 85 days vs. 22 to 28 days, unpaired t-test

^§^ p<0.01, for titers at 64 to 85 days vs. 22 to 28 days, unpaired t-test
